# Lower-limb joint hypermobility and associations with physical performance in school-aged children: a cross-sectional study

**DOI:** 10.1007/s00431-026-07469-2

**Published:** 2026-10-02

**Authors:** Blanca Mateos Blazquez, Paula Cobos Moreno, Beatriz Gómez Martín

**Affiliations:** Departamento de Enfermería, Universidad de Extremadura, Av/Virgen del Puerto, 2, Plasencia, 10600 Cáceres, España

**Keywords:** Lower-limb joint hypermobility, Physical performance, School-aged children, Youth Fitness International Test, Cross-sectional study

## Abstract

Lower-limb joint hypermobility has been associated with alterations in physical function; however, its independent relationship with physical performance in asymptomatic school-aged children remains unclear. This study investigated the association between lower-limb joint hypermobility and physical performance and determined whether these associations persisted after adjustment for potential confounding variables. A cross-sectional study was conducted in 165 school-aged children aged 4–13 years. Lower-limb joint hypermobility was assessed using the Lower Limb Assessment Score (LLAS). Participants were classified as non-hypermobile (LLAS < 7; *n* = 120) or hypermobile (LLAS ≥ 7; *n* = 45). Physical performance was evaluated using the Youth Fitness International Test (YFIT) battery, including the 20-m sprint, handgrip strength, and standing long jump. Group comparisons were performed using the Mann–Whitney *U* test, and multivariable linear regression analyses adjusted for age, sex, BMI-for-age standard deviation score (BMI-SDS), and weekly sports participation frequency were conducted. In the unadjusted analyses, children with lower-limb joint hypermobility demonstrated significantly slower 20-m sprint times (median [IQR]: 5.40 [4.90–6.10] vs. 5.00 [4.48–5.47] s; *p* = 0.005; *r* = 0.22), lower handgrip strength (9.80 [6.65–14.40] vs. 13.70 [10.40–16.75] kg; *p* < 0.001; *r* = 0.28), and shorter standing long jump distances (1.19 [1.00–1.32] vs. 1.30 [1.15–1.46] m; *p* = 0.002; *r* = 0.24). However, lower-limb joint hypermobility was not independently associated with any physical performance outcome after adjustment for age, sex, BMI-SDS, and weekly sports participation frequency (20-m sprint: *p* = 0.861; handgrip strength: *p* = 0.868; standing long jump: *p* = 0.389).

*Conclusion*: Lower-limb joint hypermobility was associated with poorer physical performance in unadjusted analyses but was not an independent determinant of physical performance after accounting for demographic, anthropometric, and lifestyle factors. Longitudinal studies are needed to clarify these associations throughout childhood.

**Table:** 

**What is Known:** • *Joint hypermobility is common in children and may be associated with musculoskeletal symptoms and functional limitations.* •*Previous studies have reported inconsistent findings regarding the relationship between joint hypermobility and physical activity levels.*	
**What is New:** *• Children with lower-limb joint hypermobility demonstrated poorer physical performance in unadjusted analyses.* •*After adjustment for age, sex, BMI SDS and sports participation frequency, lower-limb joint hypermobility was not independently associated with physical performance.* •*Demographic and lifestyle factors explained a substantial proportion of the variability in physical performance.*

**Supplementary Information:**

The online version contains supplementary material available at https://doi.org/10.1007/s00431-026-07469-2.

## Introduction

Generalized joint hypermobility (GJH) is a musculoskeletal condition characterized by a joint range of motion that exceeds the expected physiological limits for an individual’s age, sex, and biological characteristics as a consequence of increased ligamentous and capsular laxity [1, 2]. During childhood, the prevalence of joint hypermobility varies considerably depending on the diagnostic criteria applied, the assessment methods used, and the characteristics of the populations studied [1, 3]. Currently, joint hypermobility is recognized as part of a clinical spectrum ranging from asymptomatic generalized joint hypermobility to hypermobility spectrum disorders (HSD) and hypermobile Ehlers–Danlos syndrome (HEDS), allowing a more comprehensive understanding of its clinical manifestations and functional consequences [2, 4].

Although many children with joint hypermobility remain asymptomatic, others develop musculoskeletal pain, fatigue, reduced exercise tolerance, and functional limitations that interfere with daily activities and sports participation [5–7]. Previous studies have also suggested that pain, fear of movement, and reduced confidence in physical abilities may contribute to lower participation in physical activity and poorer functional outcomes during childhood and adolescence [5, 8–10]. Moreover, longitudinal studies have shown that symptomatic hypermobility may negatively influence quality of life and habitual physical activity patterns, particularly during critical periods of musculoskeletal development [11–13].

From a biomechanical perspective, lower-limb joint hypermobility is of particular interest because the foot and ankle play a fundamental role in postural stability, load transmission, and locomotor efficiency. Increased ligamentous laxity may alter lower-limb biomechanics through excessive subtalar pronation, increased mobility of the midfoot, and reduced passive joint stability, potentially increasing the demands placed on neuromuscular control during functional activities [4]. These biomechanical adaptations have been proposed to impair movement efficiency and contribute to reduced physical performance, particularly during tasks requiring speed, strength, and explosive power.

Despite the growing body of literature on generalized joint hypermobility, important knowledge gaps remain. Most previous studies have investigated generalized joint hypermobility or hypermobility spectrum disorders and have primarily included symptomatic children or adolescents with musculoskeletal complaints [2, 5, 10–13]. Consequently, relatively little is known about the functional implications of lower-limb-specific joint hypermobility in otherwise healthy and asymptomatic schoolchildren. Furthermore, previous investigations have generally relied on unadjusted comparisons and have rarely accounted for important confounding factors such as age, sex, body size, and habitual sports participation, all of which strongly influence physical performance during childhood. Therefore, whether lower-limb joint hypermobility is independently associated with physical performance remains uncertain.

The Lower Limb Assessment Score (LLAS) is a validated clinical instrument specifically developed to quantify lower-limb joint hypermobility through a standardized assessment of the hip, knee, ankle, subtalar, midtarsal, and first metatarsophalangeal joints [14, 15]. Likewise, physical performance can be objectively assessed using the Youth Fitness International Test (YFIT)**,** an internationally standardized battery developed through a Delphi consensus involving 169 experts from 50 countries and territories, which includes the 20-m sprint, handgrip strength, and standing long jump as core measures of health-related physical fitness in children and adolescents [16].

Therefore, the aim of this study was to investigate the association between lower-limb joint hypermobility and physical performance in school-aged children and to determine whether lower-limb joint hypermobility was independently associated with physical performance after adjustment for age, sex, BMI SDS, and weekly sports participation frequency. We hypothesized that children with lower-limb joint hypermobility would demonstrate poorer physical performance, characterized by longer 20-m sprint times, lower handgrip strength, and shorter standing long jump distances, compared with non-hypermobile children.

## Methods

### Study design

An analytical cross-sectional study was conducted to investigate the association between lower-limb joint hypermobility, physical performance, and physical activity levels in schoolchildren. Because of its cross-sectional design, all study variables were assessed at a single time point, allowing the identification of associations between variables without establishing causal relationships.

### Population and sample

This analytical cross-sectional study was conducted between April and July 2025 during regular school hours at a private school located in Lomas de Chapultepec, Mexico City, Mexico.

Children were eligible if they were between 4 and 13 years of age, were enrolled at the participating school, and were able to complete all study assessments. Participants were excluded if they presented an acute musculoskeletal injury at the time of assessment, had a previously diagnosed neurological or musculoskeletal disorder, were receiving treatment that could affect musculoskeletal function or physical performance, or presented any medical condition that could interfere with safe participation in the physical fitness assessments. Medical exclusion criteria were assessed based on information provided by parents or legal guardians and on clinical findings identified by the podiatrist during the study assessment.

**Fig. 1:**
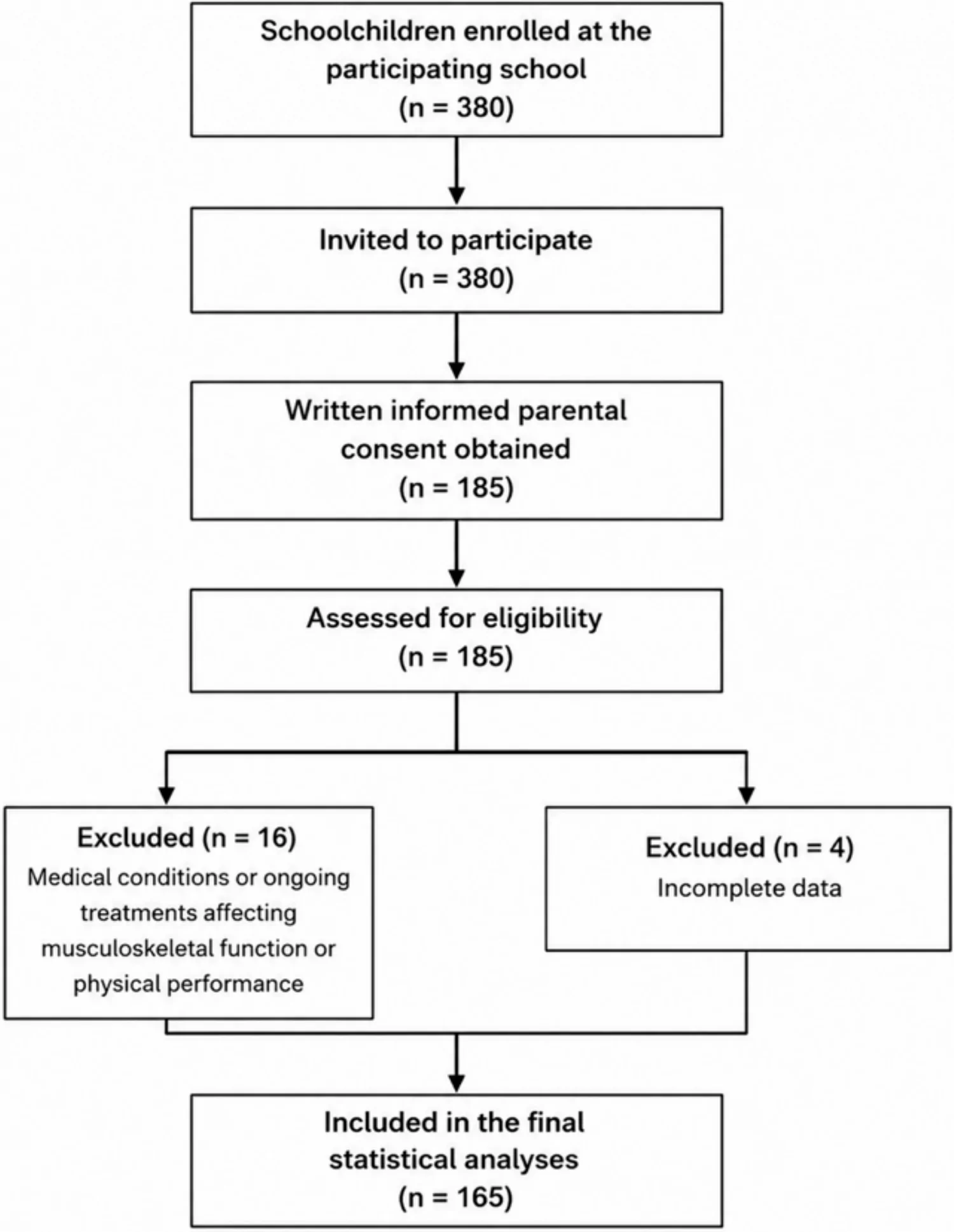
Flow diagram of participant recruitment, eligibility assessment, exclusions, and inclusion in the final statistical analyses

Approximately 380 schoolchildren enrolled at the participating school were invited to participate. Written informed consent was obtained from the parents or legal guardians of 185 children, who were subsequently assessed for eligibility. Sixteen children were excluded because they presented medical conditions or were receiving treatments that could potentially affect musculoskeletal function or physical performance. An additional four participants were excluded because of incomplete data. Consequently, 165 schoolchildren fulfilled the eligibility criteria, completed the study protocol, and were included in the final statistical analyses (Fig. 1).

### Assessment procedure

Data collection was conducted between April and July 2025 during regular school hours at a private school located in Lomas de Chapultepec, Mexico City, Mexico**.** Owing to the school’s academic organization, participants were evaluated over several assessment days according to their school grade, beginning with the youngest children and progressing sequentially to the oldest age groups.

All assessments were completed during a single evaluation session within the school facilities. Clinical examinations were performed in a classroom temporarily adapted as a clinical assessment room and equipped with an examination couch and all materials required for the evaluations. Physical performance testing was conducted on the school’s sports court located adjacent to the examination room, allowing each participant to complete the entire assessment protocol consecutively during a single visit.

Participants followed the same standardized assessment sequence. Demographic and anthropometric information was first recorded, including age, participation in organized sports, weekly sports participation frequency, body weight, height, and body mass index (BMI). Foot and footwear anthropometric measurements were subsequently obtained.

Lower-limb joint hypermobility was then assessed using the Lower Limb Assessment Score (LLAS)**,** a validated clinical instrument consisting of 12 dichotomous clinical tests, each scored as 0 (absence of hypermobility) or 1 (presence of hypermobility), resulting in a total score ranging from 0 to 12 points**.** The assessment included hip flexion, hip abduction, knee hyperextension, anterior knee drawer, knee rotation, ankle dorsiflexion, anterior ankle drawer, subtalar inversion, midtarsal inversion, midtarsal abduction/adduction with dorsiflexion–plantarflexion, first metatarsophalangeal joint mobility, and excessive subtalar pronation. Both lower limbs were examined systematically, always beginning with the left limb. Participants with an LLAS score ≥7/12 were classified as presenting lower-limb joint hypermobility according to the internationally accepted cut-off proposed by Meyer et al. and supported by pediatric reliability studies of the LLAS [14, 15].

Physical performance was subsequently evaluated using the Youth Fitness International Test (YFIT) battery, a standardized battery developed through an international Delphi consensus to assess physical fitness in children and adolescents [16]. The battery included the 20-m sprint**,** handgrip strength**,** and standing long jump tests, which assess running speed, upper-limb muscle strength, and lower-limb explosive power, respectively.

Before physical performance testing, participants completed a standardized warm-up consisting of approximately 5 min of light running and dynamic mobility exercises**,** following the recommendations of the YFIT protocol. All physical performance tests were performed on the school’s sports court while participants wore their usual sports footwear. The tests were administered in the same order for all participants (20-m sprint, handgrip strength, and standing long jump).

Sprint performance was assessed over a distance of 20 m using a digital stopwatch, with one maximal trial completed by each participant. Handgrip strength was measured using a TKK hand dynamometer. Participants stood upright with the arms fully extended alongside the body without touching the trunk, following the standardized YFIT protocol. Both hands were assessed, and the highest value obtained was retained for statistical analysis. The standing long jump test was performed from a standing position behind the starting line with both feet together. Participants were instructed to jump forward as far as possible using a bilateral take-off, and the jump distance was measured from the take-off line to the nearest heel at landing. Two attempts were performed for both the handgrip strength and standing long jump tests, and the best performance was retained for analysis. A 1-min recovery period was allowed between consecutive attempts to minimize the influence of fatigue, in accordance with the YFIT protocol.

All assessments were performed by a single podiatrist with more than 10 years of clinical experience in musculoskeletal assessment, following standardized procedures throughout the study. The same examiner performed both the LLAS assessment and the physical performance tests. Data were recorded using standardized data collection forms specifically designed for the study to ensure consistency and minimize recording errors. Each complete evaluation required approximately 40 min, although the duration varied slightly according to the participant’s age and level of cooperation.

### Ethical considerations

The study was conducted in accordance with the ethical principles of the Declaration of Helsinki and was approved by the Ethics Committee of the University of Extremadura (protocol code Nº 98/2025, approved on 3 June 2025). Information confidentiality was guaranteed through alphanumeric coding of participants. Informed consent was obtained from parents or legal guardians, and assent was obtained from all participating minors prior to participation.

### Statistical analysis

Statistical analyses were performed using IBM SPSS Statistics (IBM Corp., Armonk, NY, USA). The normality of all quantitative variables was assessed using the Shapiro–Wilk test. Because continuous variables deviated significantly from a normal distribution (*p* < 0.05), non-parametric statistical methods were used for unadjusted group comparisons. Continuous variables are presented as median and interquartile range (IQR), whereas categorical variables are expressed as absolute frequencies and percentages.

Participants were classified into two groups according to the Lower Limb Assessment Score (LLAS) using the validated cut-off proposed by Meyer et al. Participants with an LLAS <7 were classified as non-hypermobile, whereas those with an LLAS ≥7 were classified as having lower-limb joint hypermobility.

Baseline differences between non-hypermobile participants (LLAS <7) and participants with lower-limb joint hypermobility (LLAS ≥7) were assessed using the Mann–Whitney *U* test for continuous variables and the chi-square test for categorical variables, as appropriate.

BMI-for-age standard deviation scores (BMI-SDS) were derived using sex- and age-specific pediatric growth references. Subsequently, multivariable linear regression analyses were performed to evaluate whether lower-limb joint hypermobility was independently associated with physical performance outcomes after adjustment for age, sex, BMI-SDS, and weekly sports participation frequency. Regression model assumptions were evaluated by examining linearity, residual normality, homoscedasticity, multicollinearity, independence of residuals, and influential observations. Residual normality was assessed using Q–Q plots and the Shapiro–Wilk test, independence of residuals using the Durbin–Watson test, multicollinearity using variance inflation factors (VIF) and tolerance values, and potentially influential observations using Cook’s distance. Residual plots were visually inspected to assess linearity and homoscedasticity. Regression coefficients (*B*), standardized coefficients (*β*), 95% confidence intervals (95% CI), and p-values were reported. Statistical significance was established at *p* < 0.05.

Participants with incomplete data for any exposure, outcome, or covariate were excluded from the corresponding analyses. Therefore, complete-case analysis was performed.

## Results

### Baseline characteristics

A total of 165 school-aged children were included in the final analysis. The participant selection process is presented in Fig. 1. According to the Lower Limb Assessment Score (LLAS), 120 participants (72.7%) were classified as non-hypermobile (LLAS < 7), whereas 45 (27.3%) were classified as presenting lower-limb joint hypermobility (LLAS ≥ 7). Baseline demographic and anthropometric characteristics according to LLAS classification are summarized in Table 1.

**Table 1:** Baseline demographic and anthropometric characteristics according to Lower Limb Assessment Score (LLAS) classification

Variable	LLAS < 7 (*n* = 120)	LLAS ≥ 7 (*n* = 45)	*p*-value
Age (years)	9 (8–11)	8 (5–9)	< 0.001
Sex, n (%)	Female: 60 (50.0%) Male: 60 (50.0%)	Female: 27 (60.0%) Male: 18 (40.0%)	0.252^1^
Weight (kg)	30.7 (25,1–37,8)	24.2 (18,2–28,1)	< 0.001
Height (m)	1.37 (1,30–1,48)	1.26 (1,10–1,34)	< 0.001
BMI for age SDS	− 0.14 (− 0.83–0.62)	− 0.63 (− 1.39 to − 0.02)	0.007
Weekly Sports frequency (hours/week)	4 (2–7)	3 (2–4)	0.021

^1^Pearson’s chi-square test. Continuous variables are presented as median (IQR) and were compared using the Mann–Whitney *U* test

Compared with non-hypermobile participants, children with lower-limb joint hypermobility were significantly younger and had significantly lower body weight, height, and body mass index (BMI) (all *p* < 0.001). Weekly sports participation frequency was also significantly lower in the hypermobile group (*p* = 0.021). No significant differences were observed in sex distribution between the two groups (*p* = 0.252).

### Physical performance according to LLAS classification

The unadjusted comparisons of physical performance outcomes according to LLAS classification are presented in Table 2. Children classified as having lower-limb joint hypermobility (LLAS ≥ 7) required significantly more time to complete the 20-m sprint than non-hypermobile participants (median [IQR]: 5.40 [4.86–6.12] vs. 5.00 [4.48–5.47] s; Mann–Whitney *U* = 1928.0; *p* = 0.005; *r* = 0.22). Similarly, children with lower-limb joint hypermobility demonstrated significantly lower handgrip strength (9.80 [6.65–14.40] vs. 13.70 [10.40–16.75] kg; Mann–Whitney *U* = 1729.0; *p* < 0.001; *r* = 0.28) and achieved significantly shorter standing long jump distances (1.190 [1.00–1.32] vs. 1.295 [1.15–1.46] m; Mann–Whitney *U* = 1866.0; *p* = 0.002; *r* = 0.24) than children without lower-limb joint hypermobility. The observed effect sizes ranged from small to moderate. These analyses were unadjusted and therefore did not account for potential confounding variables such as age, sex, body mass index, or weekly sports participation frequency. Adjusted multivariable regression analyses are presented in Table 3.

**Table 2:** Unadjusted comparisons of physical performance according to lower-limb joint hypermobility

Outcome	LLAS < 7 (***n*** = 120) Median (IQR)	LLAS ≥ 7 (***n*** = 45) Median (IQR)	Mann–Whitney ***U***	***Z***	Effect size (***r***)	***p***-value
20-m sprint (s)	5.00 (4.48–5.47)	5.40 (4.86–6.12)	1928	− 2.825	0.22	0.005
Handgrip strength (kg)	13.70 (10.40–16.75)	9.80 (6.65–14.40)	1729	− 3.553	0.28	< 0.001
Standing long jump (m)	1.29 (1.15–1.46)	1.19 (1.00–1.32)	1866	− 3.053	0.24	0.002

Effect size (*r*) was calculated as |Z|/√N and interpreted according to Cohen’s criteria (0.10 = small, 0.30 = moderate, 0.50 = large)

**Table 3:** Multivariable linear regression models evaluating the independent association between lower-limb joint hypermobility (LLAS ≥ 7) and physical performance outcomes

Predictor	20-m Sprint B (95% CI)	***β***	*p*	Handgrip Strength B (95% CI)	***β***	*p*	Standing Long Jump B (95% CI)	***β***	*p*
Lower-limb hypermobility (LLAS ≥ 7)	− 0.025 (− 0.303 to 0.253)	− 0.011	0.861	− 0.118 (− 1.521 to 1.284)	− 0.009	0.868	− 0.035 (− 0.114 to 0.045)	− 0.059	0.389
Age (years)	− 0.234 (− 0.288 to − 0.181)	− 0.575	< 0.001	1.880 (1.610 to 2.150)	0.756	< 0.001	0.062 (0.046 to 0.077)	0.559	< 0.001
BMI-SDS	0.030 (− 0.070 to 0.129)	0.037	0.553	0.513 (0.011 to 1.014)	0.103	0.045	− 0.011 (− 0.039 to 0.017)	− 0.049	0.449
Weekly sports participation (hours/week)	− 0.053 (− 0.087 to − 0.019)	− 0.195	0.003	0.030 (− 0.142 to 0.203)	0.018	0.729	0.007 (− 0.002 to 0.017)	0.101	0.135
Sex	0.083 (− 0.150 to 0.315)	0.043	0.483	− 0.078 (− 1.249 to 1.093)	− 0.007	0.895	0.005 (− 0.061 to 0.071)	0.009	0.889
Model R^2^	0.442			0.619			0.382		
Adjusted R^2^	0.424			0.607			0.363		
*F*	25.2			51.7			19.7		
Model *p*-value	< 0.001			< 0.001			< 0.001		

*B* unstandardized regression coefficient, *β* standardized regression coefficient, *CI* confidence interval, *BMI-SDS* body mass index-for-age standard deviation score, *LLAS* Lower Limb Assessment Score. Sex was coded as 0 = female and 1 = male. All regression models were adjusted for age, sex, BMI-SDS, and weekly sports participation frequency. Model *F* statistics were *F*(5,159) = 25.2 for the 20-m sprint, *F*(5,159) = 51.7 for handgrip strength, and *F*(5,159) = 19.7 for the standing long jump

### Multivariable analyses

Multivariable linear regression analyses adjusted for age, sex, BMI-for-age standard deviation score (BMI-SDS), and weekly sports participation frequency are presented in Table 3. All three regression models were statistically significant (20-m sprint: R^2^ = 0.442, adjusted R^2^ = 0.424, *F*(5,159) = 25.2, *p* < 0.001; handgrip strength: R^2^ = 0.619, adjusted R^2^ = 0.607, *F*(5,159) = 51.7, *p* < 0.001; standing long jump: R^2^ = 0.382, adjusted R^2^ = 0.363, *F*(5,159) = 19.7, *p* < 0.001). However, lower-limb joint hypermobility (LLAS ≥ 7) was not independently associated with any physical performance outcome after adjustment for the included covariates (20-m sprint: *p* = 0.861; handgrip strength: *p* = 0.868; standing long jump: *p* = 0.389).

Age was significantly associated with all three physical performance outcomes (all *p* < 0.001), with increasing age associated with faster 20-m sprint times, greater handgrip strength, and longer standing long jump distances. BMI-SDS was positively associated with handgrip strength (*B* = 0.513, 95% CI: 0.011 to 1.014; *β* = 0.103; *p* = 0.045) but was not significantly associated with 20-m sprint (*p* = 0.553) or standing long jump performance (*p* = 0.449). Weekly sports participation frequency was independently associated with faster 20-m sprint times (*B* =  − 0.053, 95% CI: − 0.087 to − 0.019; *β* =  − 0.195; *p* = 0.003), but not with handgrip strength or standing long jump performance. Sex was not independently associated with any of the physical performance outcomes.

Model diagnostics showed no evidence of problematic multicollinearity (VIF range: 1.04–1.26; tolerance range: 0.791–0.962), and no highly influential observations were identified based on Cook’s distance (maximum values: 0.286, 0.237, and 0.063 for the 20-m sprint, handgrip strength, and standing long jump models, respectively). Residuals did not significantly deviate from normality for the standing long jump model (Shapiro–Wilk *W* = 0.985, *p* = 0.077), whereas significant deviations were observed for the 20-m sprint (*W* = 0.957, *p* < 0.001) and handgrip strength models (*W* = 0.872, *p* < 0.001). The Durbin–Watson statistics were 1.72 (*p* = 0.044), 2.08 (*p* = 0.678), and 2.04 (*p* = 0.810) for the 20-m sprint, handgrip strength, and standing long jump models, respectively.

## Discussion

The present study investigated the association between lower-limb joint hypermobility and physical performance in school-aged children using both unadjusted and multivariable analyses. In the unadjusted analyses, children with lower-limb joint hypermobility demonstrated poorer performance in sprint speed, handgrip strength, and standing long jump compared with non-hypermobile participants. However, these associations were no longer significant after adjustment for age, sex, BMI-for-age standard deviation score (BMI-SDS), and weekly sports participation frequency.

The unadjusted findings are partially consistent with previous studies reporting reduced physical performance among children and adolescents with generalized joint hypermobility or hypermobility spectrum disorders. Juul-Kristensen et al. [3] reported impairments in motor competence, coordination, and postural control in children with generalized joint hypermobility, although they did not observe significant differences in habitual physical activity. Similarly, Engelbert et al. [6] demonstrated reduced exercise tolerance and increased fatigue in children with joint hypermobility syndrome, whereas Van Meulenbroek et al. [8] described lower muscle strength and greater perceived vulnerability to injury among hypermobile adolescents. Schubert-Hjalmarsson et al. [5] also reported impairments in balance, participation, and motor performance in children with hypermobility syndrome. More recently, Ituen et al. [11] showed that generalized joint hypermobility negatively influences quality of life and physical activity trajectories during childhood, reinforcing the clinical relevance of early identification and follow-up.

An important contribution of the present study is that the significant differences observed in the unadjusted analyses disappeared after controlling for potential confounding variables. Age was the strongest independent predictor across all physical performance outcomes. BMI-SDS was positively associated with handgrip strength but was not independently associated with 20-m sprint or standing long jump performance, whereas weekly sports participation frequency was independently associated only with 20-m sprint performance. These findings indicate that age-related development, body size relative to age and sex, and habitual participation in sport contribute differently to specific components of physical performance during childhood. Consequently, studies based exclusively on unadjusted comparisons may overestimate the independent contribution of joint hypermobility to physical performance. These findings also highlight the importance of considering potential confounding variables when investigating physical performance in pediatric populations with lower-limb joint hypermobility.

The discrepancies between the present findings and some previous studies may also be explained by differences in the populations evaluated. Most published investigations have focused on children or adolescents with generalized joint hypermobility, hypermobility spectrum disorders, or hypermobile Ehlers–Danlos syndrome, conditions that are frequently accompanied by pain, fatigue, reduced physical conditioning, and functional limitations [2, 5, 6, 10, 12, 13]. In contrast, the present study evaluated lower-limb joint hypermobility using the LLAS in a sample of schoolchildren from the general population, most of whom were asymptomatic. Therefore, lower-limb joint hypermobility itself may not be sufficient to impair physical performance unless accompanied by additional clinical manifestations or associated disorders. Because lower-limb joint hypermobility and physical performance were assessed simultaneously, the temporal direction of the observed associations cannot be established. Consequently, reverse causation cannot be excluded, and longitudinal studies are required to clarify the temporal relationship between lower-limb joint hypermobility and physical performance.

From a clinical perspective, these findings suggest that lower-limb joint hypermobility alone should not be considered an isolated determinant of impaired physical performance in children. Instead, clinicians should perform comprehensive assessments that incorporate age-related developmental factors, anthropometric characteristics, habitual physical activity, and the presence of pain or other musculoskeletal symptoms when evaluating children with lower-limb joint hypermobility. This multidimensional approach may facilitate the identification of individuals who could benefit most from targeted interventions aimed at improving muscle strength, neuromuscular control, proprioception, and long-term participation in physical activity [6, 10].

The present study has several strengths. It included a relatively large sample of schoolchildren evaluated using standardized procedures by a single experienced examiner, thereby minimizing measurement variability. Furthermore, validated instruments were used to assess lower-limb joint hypermobility (LLAS) and physical performance (YFIT), and multivariable regression analyses enabled adjustment for important confounding variables, providing a more robust interpretation of the association between lower-limb joint hypermobility and physical performance.

Some limitations should also be acknowledged. First, the cross-sectional design precludes establishing causal relationships between lower-limb joint hypermobility and physical performance. Second, participants were recruited from a single private school in Mexico City, which may limit the generalizability of the findings to other pediatric populations and educational settings. Third, no formal *a priori* sample size calculation was performed because the study included all eligible children who met the inclusion criteria during the recruitment period. Furthermore, although only a limited number of predefined outcomes were analyzed, multiple statistical comparisons may have increased the probability of type I error. Therefore, statistically significant findings should be interpreted with appropriate caution. Finally, potentially relevant variables such as biological maturation, pain intensity, muscle endurance, and psychosocial factors were not evaluated and may also influence physical performance in children with joint hypermobility [7, 9].

In conclusion, lower-limb joint hypermobility was associated with poorer physical performance in the unadjusted analyses; however, these associations were no longer significant after adjustment for age, sex, BMI-for-age standard deviation score (BMI-SDS), and weekly sports participation frequency. These findings suggest that lower-limb joint hypermobility alone is not an independent determinant of physical performance in school-aged children. Clinical assessment should therefore consider demographic, anthropometric, and lifestyle factors when evaluating physical function in children with lower-limb joint hypermobility. Future longitudinal studies are warranted to clarify the temporal relationship between lower-limb joint hypermobility, growth and maturation, and physical performance and to explore potential dose–response relationships using the continuous LLAS score.

## Supplementary Information

Below is the link to the electronic supplementary material.

ESM 1 (PDF 83.3 KB)

## Data Availability

The datasets generated and analyzed during the current study are not publicly available due to privacy and ethical restrictions regarding the pediatric participants but are available from the corresponding author on reasonable request.
