# Supplementary material for: Lower-limb joint hypermobility and associations with physical performance in school-aged children: a cross-sectional study
Source: Eur J Pediatr. 2026 Oct 2;185(11):798. doi: 10.1007/s00431-026-07469-2 (PMC13633312; doi:10.1007/s00431-026-07469-2)
Supplement: Supplementary file 1 [file 431_2026_7469_MOESM1_ESM.pdf]

Item	Recommendation	Page No.
**Title and abstract**		
**1(a)**	Indicate the study's design with a commonly used term in the title or the abstract	1
**1(b)**	Provide in the abstract an informative and balanced summary of what was done and what was found	1
**Introduction**		
**2**	Explain the scientific background and rationale for the investigation	2–3
**3**	State specific objectives, including any prespecified hypotheses	3
**Methods**		
**4**	Present key elements of study design early in the paper	3
**5**	Describe the setting, locations, and relevant dates, including periods of recruitment, exposure, follow-up, and data collection	3–4
**6(a)**	Give the eligibility criteria and the sources and methods of selection of participants	3–4
**7**	Clearly define all outcomes, exposures, predictors, potential confounders, and effect modifiers	4–6
**8***	For each variable of interest, give sources of data and details of methods of assessment (measurement)	4–6
**9**	Describe any efforts to address potential sources of bias	6
**10**	Explain how the study size was arrived at	3–4
**11**	Explain how quantitative variables were handled in the analyses	6
**12(a)**	Describe all statistical methods, including those used to control for confounding	6–7
**12(b)**	Describe any methods used to examine subgroups and interactions	N/A
**12(c)**	Explain how missing data were addressed	7
**12(d)**	If applicable, describe analytical methods taking account of sampling strategy	N/A
**12(e)**	Describe any sensitivity analyses	N/A
**Results**		
**13(a)**	Report numbers of individuals at each stage of study	7
**13(b)**	Give reasons for non-participation at each stage	Figure 1
**13(c)**	Consider use of a flow diagram	Figure 1
**14(a)**	Give characteristics of study participants	7–8
**14(b)**	Indicate number of participants with missing data for each variable	7

**15**	Report numbers of outcome events or summary measures	8–10
**16(a)**	Give unadjusted estimates and, if applicable, confounder-adjusted estimates and their precision	8–10
**16(b)**	Report category boundaries when continuous variables were categorized	5–6
**16(c)**	If relevant, consider translating estimates of relative risk into absolute risk	N/A
**17**	Report other analyses done	9–10
**Discussion**		
**18**	Summarise key results with reference to study objectives	10
**19**	Discuss limitations of the study	12
**20**	Give a cautious overall interpretation of results	10–13
**21**	Discuss the generalisability (external validity) of the study results	12
**Other information**		
**22**	Give the source of funding and the role of the funders	Funding / Declarations
